# MOFU: Development of a MOrphing Fluffy Unit with expansion and contraction capabilities and evaluation of the animacy of its movements

**DOI:** 10.1371/journal.pone.0354179

**Published:** 2026-08-18

**Authors:** Taisei Mogi, Mari Saito, Yoshihiro Nakata

**Affiliations:** 1 The Department of Mechanical and Intelligent Systems Engineering, Graduate School of Informatics and Engineering, The University of Electro-Communications, Chofu, Tokyo, Japan; 2 Sony Corporation, Sony City Osaki, Osaki, Shinagawa-ku, Tokyo, Japan; CINVESTAV IPN: Centro de Investigacion y de Estudios Avanzados del Instituto Politecnico Nacional, MEXICO

## Abstract

Robots designed for therapy and social interaction are often intended to evoke a sense of “animacy” in humans. While many robots have been developed to imitate life-like appearance and joint movements, relatively little attention has been given to the effect of whole-body expansion and contraction—volume-changing movements commonly observed in living organisms—on perceived animacy. In this study, we developed MOFU (MOrphing Fluffy Unit), a mobile robot capable of whole-body expansion and contraction using a single motor enclosed within a fluffy exterior. MOFU employs a “Jitterbug” structure, a geometric transformation mechanism that enables smooth volume changes using a single actuator, with the robot height varying from approximately 210 mm to 280 mm. Additionally, it is equipped with a differential two-wheel drive mechanism for locomotion. We conducted an online survey using videos of MOFU’s behavior to evaluate the effect of expansion–contraction movements on animacy perception. Participants rated their impressions using the Godspeed Questionnaire Series. First, we compared participants’ ratings across videos of MOFU in stationary states, with and without expansion–contraction, and separately, with and without rotational motion. Both the expansion–contraction and rotational movements independently increased perceived animacy. Second, we hypothesized that presenting two MOFUs simultaneously would enhance perceived animacy further; however, this prediction was not supported, as no significant difference was observed. Exploratory analyses were also performed across the four dual-robot motion conditions. Third, when rotational and expansion–contraction motions were combined with locomotion, animacy ratings were higher than for locomotion alone. These results suggest that volume-changing movements, such as expansion and contraction, enhance the perception of animacy in robots. Consequently, incorporating physical volume change may be an important design element in the development of future robots aimed at shaping human impressions.

## Introduction

Robots designed for therapy and social interaction are often intended to evoke a sense of “animacy” in users. For instance, the seal-shaped robot PARO has been employed to promote emotional engagement among elderly individuals, particularly those with dementia, and its therapeutic effects have been reported [1]. Similarly, the bear-shaped robot Huggable has been shown to reduce anxiety and stress in hospitalized children through interactive engagement [2]. These robots typically integrate elements such as animal-like appearances, soft tactile surfaces, and responsive behaviors, all of which contribute to shaping human–robot relationships.

**Fig 1:**
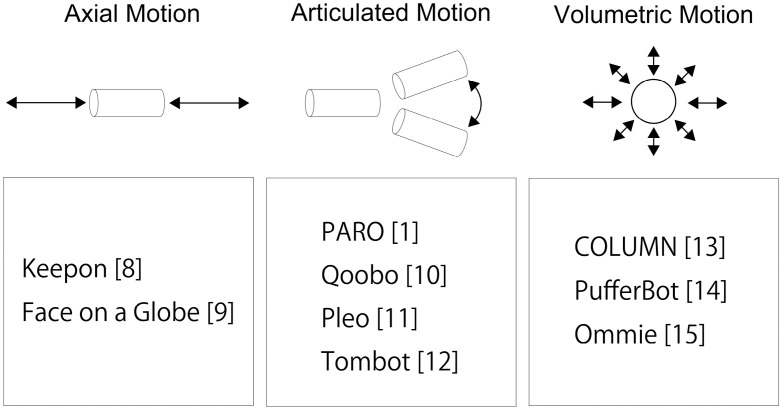
Classification of robot body motions into three categories—axial, articulated, and volumetric motion—based on structural characteristics of movement. Robots shown are Keepon [8], Face on a Globe [9], PARO [1], Qoobo [10], Pleo [11], Tombot [12], COLUMN [13], PufferBot [14], and Ommie [15].

Perceived animacy refers to the degree to which an observer attributes life-like qualities—such as agency, goal-directedness, and intentionality—to an entity through higher-level perceptual and cognitive processes, independently of whether the entity is biologically alive [3]. Wolf et al. demonstrated that the perception of animacy in humans, animals, and machines is influenced not only by appearance but also by the presence or absence of movement [4]. This finding indicates that animacy in robots is shaped not solely by external appearance but also by the nature of their movements and responses. Lifelike movements, in particular, are considered essential for robots to be perceived as intuitive and approachable. Previous studies have shown that both a robot’s appearance and the characteristics of its movements strongly affect impressions of animacy, likability, trustworthiness, and unpleasantness [5,6]. To organize the range of robot movements explored in human–robot interaction research, we classify them into three categories—axial, articulated, and volumetric motion—based on prior literature and our own analysis (Fig 1).

Axial motion encompasses linear extension and contraction, as observed in organisms such as earthworms and inchworms [7], as well as temporary retraction along the body axis, exemplified by Keepon [8]. Robots that replicate the movements of worms or inchworms have typically been developed for bio-inspired purposes, such as locomotion in confined environments or adaptation to unstructured terrain. Additionally, studies have explored smooth, unidirectional volumetric deformation to evoke lifelike impressions [9]. However, the influence of these movements on the perceived animacy has been only minimally examined. Articulated motion involves the flexion and extension of joints, as well as the movements of continuous multi-joint structures, such as a backbone or flexible body parts, such as a tail [10–12].

In contrast, volumetric motion involves changes in the overall body volume through expansion and contraction. In living organisms, this phenomenon occurs not only in physiological functions such as breathing but also in social displays, including courtship displays of peacocks and the throat pouch inflation of frigatebirds. Articulated motion, segmented and localized movements in particular, has been widely used in social and companion robots to express affective states and create life-like impressions. By incorporating volumetric motion, robots can achieve global and continuous shape changes that are challenging to realize with conventional joint-driven mechanisms. Hence, volumetric motion has the potential to significantly expand the expressive capabilities of robots.

Building on this concept, several devices have been developed that incorporate expansion–contraction movements inspired by physiological rhythms, such as breathing and heartbeat. For instance, Ommie [15] is designed to mimic breathing motions with the goal of reducing users’ anxiety and tension. These devices typically employ localized volume changes in specific parts of the body to elicit emotional effects, such as fostering comfort and empathy. In contrast, PufferBot [14] uses a whole-body expansion–contraction mechanism to support functions such as maintaining safe interpersonal distance or facilitating locomotion. However, in these cases, volumetric changes are primarily functional or behavioral; the potential of such movements to evoke animacy in humans has not been sufficiently explored.

This study aims to clarify the effect of whole-body expansion–contraction movements (volumetric motion) on the perception of animacy in robots. To this end, we designed and developed MOFU (MOrphing Fluffy Unit), a mobile robot with a “Jitterbug” structure, a geometric transformation mechanism that enables smooth volumetric change using a single actuator. MOFU uses a single motor-driven linear mechanism for expansion and contraction and a differential two-wheel drive for locomotion, all covered with a soft, fluffy exterior. A mathematical model was used to predict dimensional changes during expansion and contraction; experimental results confirmed that the robot operated as designed. To evaluate MOFU’s behavior, we conducted an online survey. Participants watched videos of MOFU and rated their impressions using the Animacy subscale of the Godspeed Questionnaire Series. We focused specifically on perceived animacy because it represents a foundational construct in human–robot interaction: it is widely regarded as a prerequisite for attributing social qualities such as intentionality, agency, and emotional responsiveness to robots [3], and is therefore particularly relevant to evaluating the social and therapeutic potential of robots. Specifically, we examined three conditions: (i) effects of expansion–contraction and rotational motions in a stationary state (single-robot condition), (ii) impressions when two MOFUs were presented simultaneously (dual-robot condition), and (iii) effects of combining rotational and expansion–contraction motions with locomotion (locomotion condition). The dual-robot condition included four motion variants analogous to those in the single-robot condition to examine generalization, with comparisons among these dual-robot variants treated as exploratory.

The rationale for each condition is as follows. The first condition (single-robot, stationary) was selected to isolate the independent contributions of EC and rotational motion to animacy ratings under controlled conditions. The choice of the locomotion condition was motivated by perceptual research showing that moving objects accompanied by shape or area changes—such as caterpillar-like deformation—are perceived as more animate than those undergoing simple linear motion [16]; accordingly, Experiment 3 examined whether adding rotational and expansion–contraction motions to locomotion would increase perceived animacy relative to locomotion alone. The dual-robot condition was chosen because prior human–robot interaction research has demonstrated that the number of robots influences people’s social responses: Shiomi et al. [17] showed that social rewards from two agents improved motor skills to a greater extent than the improvement achieved from a single agent, and Okada et al. [18] found that apologies from two robots were evaluated more favorably than from one robot.

The contributions of this study are as follows:

1. **Development of a mobile robot capable of volumetric expansion and contraction:** We designed and implemented MOFU, a mobile robot that employs a Jitterbug structure to effortlessly expand and contract its body using a single motor, with the robot height varying from approximately 210 mm to 280 mm. A mathematical model was employed to predict dimensional changes. Experiments successfully validated the robot’s performance, confirming its functionality as intended.
2. **Empirical demonstration in a single-robot condition:** By comparing conditions with and without expansion–contraction and rotational motions when stationary (i.e., without locomotion), we demonstrated that expansion–contraction significantly enhances perceived animacy.
3. **Evaluation in a dual-robot condition:** We investigated the impressions of two robots presented simultaneously; the results did not indicate an increase in animacy compared to that of a single robot presentation. The within-dual-robot comparisons among the four motion conditions were exploratory.
4. **Evaluation in a locomotion condition:** We demonstrated that when rotational and expansion–contraction motions were combined with locomotion, perceived animacy was higher than with locomotion alone.

## Design and development of MOFU

**Fig 2:**
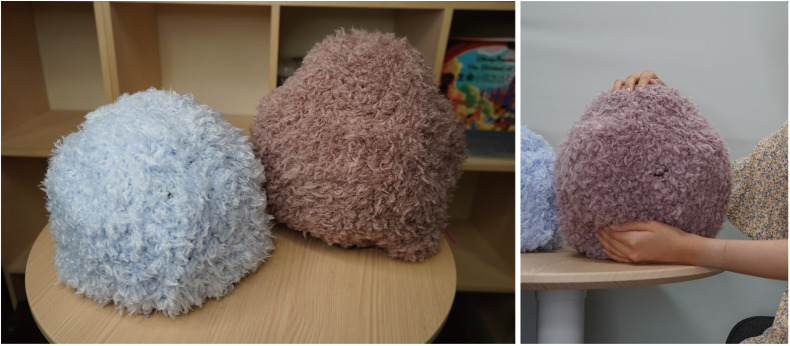
Two color variations of MOFU and a MOFU being gently stroked. MOFU was designed to evoke a sense of life-likeness through its volumetrically deformable body.

This section describes the design and implementation of the developed robot, MOFU (Fig 2).

We first outline the concept, followed by detailed explanations of the mechanical design, electrical and control systems, and the prototyping and implementation process. Next, we present the modeling and validation of the expansion–contraction mechanism, and finally, we summarize the robot motions that can be achieved with MOFU.

### Design concept

The robot developed in this study was designed around an expansion–contraction mechanism to evoke a sense of animacy, complemented by several additional features. Here, we describe the specific design concepts of quiet operation, mobility, and a fluffy exterior.

As animal muscles typically do not produce sound during movement, minimizing operational noise was considered essential for achieving a sense of “animacy.” To this end, the robot uses direct-drive motors without gears and incorporates components with low sliding noise. This design suppresses mechanical sounds during motion, reducing the perception of mechanical artifacts among users. When measured in an indoor environment with ambient noise, the sound level during both locomotion and expansion–contraction remained below 50 dB, as recorded by a sound level meter (Testo 816-1) placed approximately 50 cm from MOFU.

Animals that humans commonly interact with can move autonomously. Accordingly, the robot was equipped with a mobility mechanism. Structural simplicity and compactness were maintained by adopting wheeled locomotion. A differential two-wheel drive mechanism enables both straight movement and rotation on a horizontal plane. Wheels were selected over legs to prioritize stability, as expansion–contraction was the primary interaction element. Additionally, the use of an internal power supply and elimination of external cables enhance the robot’s autonomous appearance.

Furthermore, many animals are covered with skin or fur, which contributes to impressions of animacy. Hence, the robot was enveloped in a soft, fluffy cover to emulate this characteristic. Previous studies comparing robots of the same design but with soft versus hard surface textures have shown that soft textures can reduce psychological and physiological stress, suggesting their potential effectiveness in robot therapy [19].

The spherical form factor was selected because the underlying Jitterbug structure naturally approximates a spherical geometry during its expansion–contraction cycle. Using a spherical exterior therefore provides a geometrically consistent representation of the mechanism while allowing the volumetric deformation to be clearly expressed.

In addition, an abstract, non-animal-specific form was intentionally adopted. This design choice was made to isolate the effect of volumetric motion on perceived animacy while minimizing potential confounds arising from animal recognition because of more explicitly biomorphic shapes.

Two color variations (blue and pink) were chosen to reduce the likelihood that the observed effects would be specific to a single color condition. These colors were also selected to avoid associations with familiar animals or specific species, thereby minimizing the influence of appearance-based cues on animacy judgments. However, color itself may function as a social cue. In particular, blue and pink can be associated with binary gender stereotypes, and pink coloration may shift the perceived gender of a robot toward female [20]. Therefore, although the color variations were introduced to avoid animal-specific associations, the present design cannot exclude the possibility that color-based social categorization influenced participants’ impressions.

Finally, although biologically inspired motion patterns such as breathing rhythms could have been implemented, the expansion–contraction cycle was instead set to a constant, non-biological rate. This design decision was intended to decouple the perceptual effect of volumetric motion from additional cues associated with recognizable biological rhythms, allowing a clearer evaluation of how global body-volume changes alone influence perceived animacy.

### Mechanical design

**Fig 3:**
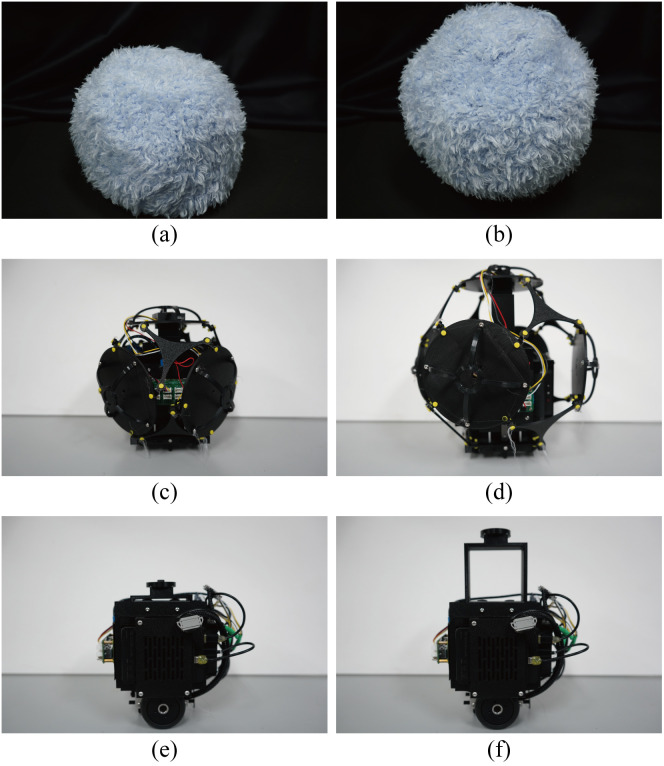
Appearance of MOFU under different conditions: (a) with fluffy cover, contracted; (b) with fluffy cover, expanded; (c) without cover, contracted; (d) without cover, expanded; (e) without mechanism, contracted; (f) without mechanism, expanded.

MOFU has a spherical shape (Fig 3) with a vertical height ranging from 210 to 280 mm, enabling whole-body expansion–contraction motion. This motion is achieved using a Jitterbug structure, where vertical displacement is controlled by a linear mechanism composed of a 20 mm lead screw (MTSPW1220, MISUMI Group Inc.) driven by a direct-drive motor (KM-1S-M4021, Keigan Inc.). The Jitterbug structure, previously implemented in the modular robot AuxBot developed by Chin et al., enables expansion–contraction of the robot’s body using a single actuator [21,22].

**Fig 4:**
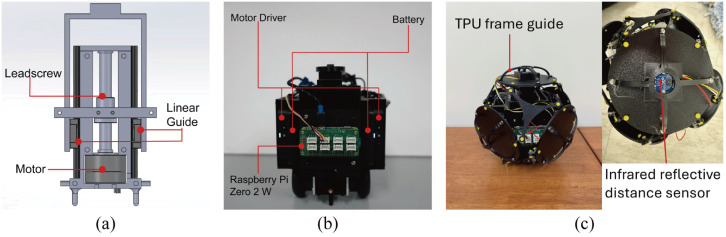
Mechanical design of MOFU: (a) Arrangement of the leadscrew and linear guides. (b) Arrangement of the motor driver, battery, and controller. (c) Appearance of TPU frame guide and the mounting position of the touch sensor.

The Jitterbug structure is inherently polyhedral; hence, 3-D printed TPU frames were attached to five faces, excluding the bottom face, to give the overall exterior a more spherical appearance. Additionally, an infrared reflective distance sensor (Grove – Infrared Reflective Sensor, Seeed Studio) was mounted on the inner side of the top frame (Fig 4c). This sensor measures the distance to the fluffy cover to detect contact. Although it was not used in the experiments reported here, it was implemented to enable potential future contact-based interactions. Similar sensors can be installed on other frames to easily expand the number of contact-detectable areas.

A quiet linear guide (TK-04, Igus K.K.) was adopted in combination with the direct-drive motor for the linear mechanism (Fig 4a) to ensure quiet operation and backdrivability, thereby minimizing operating noise. Locomotion on the horizontal plane was enabled by a differential two-wheel drive system, using two additional direct-drive motors of the same type to drive the wheels. Rubber tires (NARROW TIRE SET, 58 mm DIA., TAMIYA, INC.) suitable for the motor size were employed. Sliding contact parts were added at the front and rear of the bottom surface to prevent tipping, ensuring stability by maintaining four points of contact with the ground.

The arrangement of the individual components is illustrated in Fig 4b.

### Electrical and control systems

**Fig 5:**
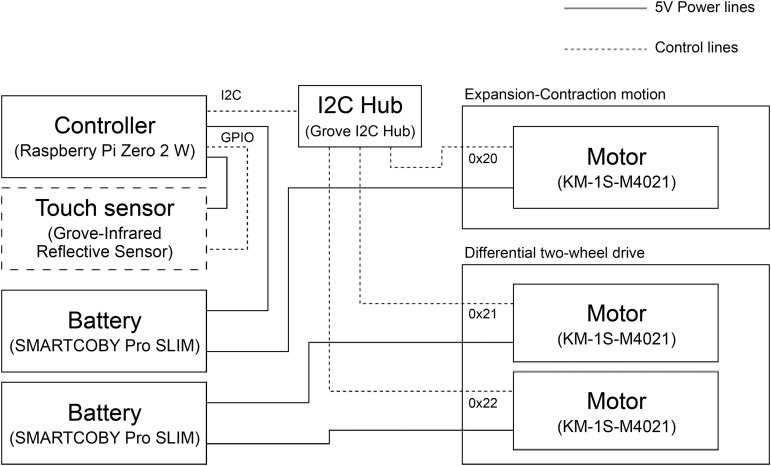
System configuration of MOFU.

The system configuration of MOFU is illustrated in Fig 5. MOFU is powered by three direct-drive motors (KM-1S-M4021, Keigan Inc.), which are controlled by motor drivers via feedback according to position commands provided by a controller (Raspberry Pi Zero 2 W, Raspberry Pi Ltd). The PID control gains were set to $K_{p}=5.0,K_{i}=10.0,K_{d}=0$. Power for both the motor drivers and the controller is supplied by two lithium-ion batteries (SMARTCOBY Pro SLIM, CIO Co., Ltd.), each with a capacity of 10,000 mAh and an output voltage of 5 V. As mentioned earlier, an infrared reflective distance sensor is installed on the top surface of MOFU, although it was not used in the experiments reported in this study. This sensor provides a binary signal indicating whether the top surface is in contact, which the controller can use to trigger motions or other interactions.

MOFU can also detect collisions and perform responsive actions to contact with humans or walls by utilizing the wheel torque sensor and setting an appropriate threshold (0.08 N m).

### Prototyping and implementation

The main specifications of MOFU are listed in Table 1. Key parameters, including the vertical height range and total weight, were measured and confirmed. These specifications were used to assemble the prototype by integrating the mechanical, electrical, and control systems. Additionally, the wheels were wrapped with yarn (SKI YARN Tenshi no Fur, Motohiro & Co., Ltd.) to match the fluffy exterior and further reduce mechanical impressions.

**Table 1:** Main specifications of MOFU.

Item	Value	Measurement condition/method
Overall height (including wheels and frame)	210–280 mm	Measured, maximum vertical point at rest
Distance between wheel contact points	90 mm	Distance between ground contact points of the wheels
Wheel diameter	58 mm	Rubber wheel diameter
Total battery capacity	20000 mAh	–
Total weight	2091 g	Including batteries and exterior cover

S1 Fig shows the internal components of MOFU.

### Modeling and validation of the expansion–contraction mechanism

As mentioned earlier, the expansion–contraction motion of MOFU is based on the Jitterbug structure, which naturally produces rotational motion during deformation. This apparent rotation is minimized by the differential two-wheel drive, which compensates for the induced rotation, enabling MOFU to appear to expand and contract independently.

The model equation was constructed based on the Jitterbug model proposed by Verheyen et al. [23], with reference to the work of Chin et al. [21]. The vertical displacement *Z* of the Jitterbug structure is expressed as a function of the rotation angle Θ of the top face relative to the base as follows:


$$\begin{array}{c} Z(\Theta )=2\sqrt{r_{x}^{2}+r_{y}^{2}+r_{z}^{2}}+C \end{array}$$ (1)

where $r_{x}$, $r_{y}$, and $r_{z}$ are defined as


$$\begin{array}{c} r_{x}(\mu )=R_{A}\cos\mu \end{array}$$ (2)

$$\begin{array}{c} r_{y}(\mu )=R_{A}\sin\mu \end{array}$$ (3)

$$\begin{array}{c} r_{z}(\mu )=\frac{R_{A}\cos\theta_{dh}\cos\mu +\sqrt{R_{B}^{2}-R_{A}^{2}\sin^{2}\mu }}{\sin\theta_{dh}} \end{array}$$ (4)

Here, $R_{A}$, $R_{B}$, and $\theta_{dh}$ are geometric parameters of the Jitterbug structure. Furthermore, μ is defined as


$$\begin{array}{c} \mu =\mu_{0}-\theta \end{array}$$ (5)

where $\mu_{0}$ is calculated from $R_{A}$ and $R_{B}$ as follows:


$$\begin{array}{c} \mu_{0}=\arcsin\frac{R_{B}}{R_{A}} \end{array}$$ (6)

The angle Θ is then related to θ as


$$\begin{array}{c} \Theta =2\theta \end{array}$$ (7)

**Fig 6:**
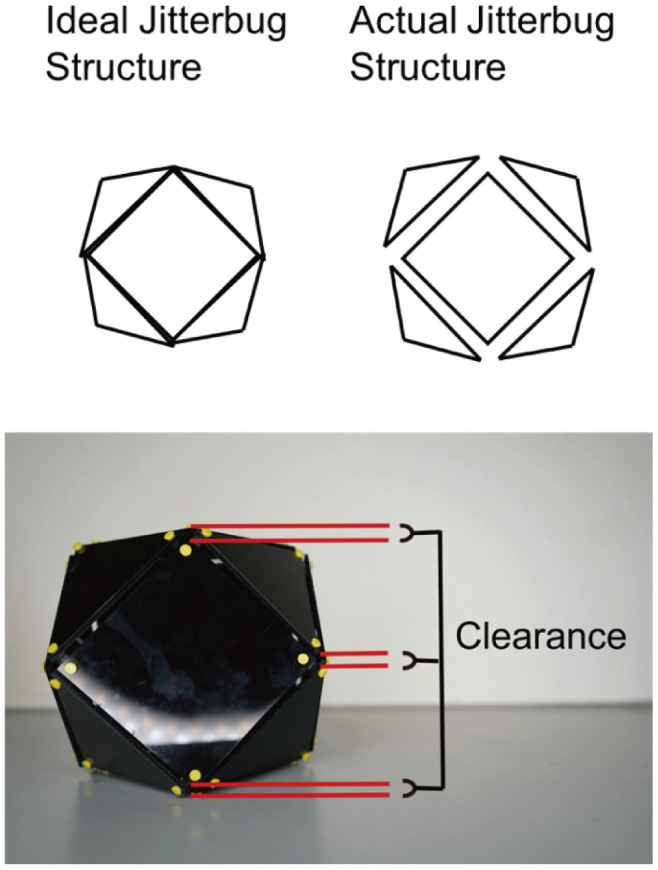
Gap generated by the joints of the Jitterbug structure.

Thus, once Θ is determined, *Z* is uniquely obtained. In Eq. 1, the term *C* represents a clearance constant specific to MOFU’s Jitterbug structure. Although an ideal Jitterbug structure has no gaps between its faces, MOFU’s joints introduce mechanical clearances (Fig 6). This clearance is represented by the constant *C*. The values of $R_{A}$, $R_{B}$, $\theta_{dh}$, and *C* are summarized in Table 2.

**Table 2:** Geometric parameters for the Jitterbug structure in MOFU.

Parameter	Description	Value
$R_{A}$	Radius associated with geometry A	$80\sqrt{2}/2=56.6$ mm
$R_{B}$	Radius associated with geometry B	$80\sqrt{3}/3=46.2$ mm
$\theta_{dh}$	Dihedral angle of the Jitterbug	$54.74^{\circ }$ (0.956 rad)
*C*	Clearance constant	13 mm

In practice, *Z* is used as the control target for MOFU’s expansion–contraction motion. Therefore, the inverse function of Eq. 1 has to be computed. However, a direct inverse calculation is not feasible because Eq. 1 is highly complex. Instead, the Jitterbug angle Θ was sampled at 45 evenly spaced points in the range 0−1.0 rad, and the corresponding *Z* values were precomputed. During control, when a target *Z* was given, the corresponding Θ was selected from this table.

**Fig 7:**
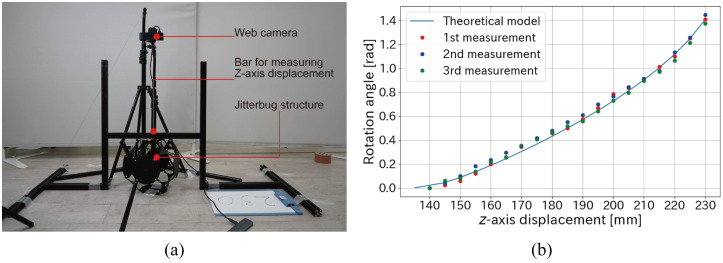
Measurement of the Jitterbug structure. (a) Experimental setup. (b) Comparison between the model curve and measurement results.

To validate the model, we experimentally examined the relationship between the vertical displacement *Z* and the rotation angle Θ of the Jitterbug structure. A webcam and ArUco markers [24] were used to measure the rotation angle, while the vertical displacement was measured three times using a jig. The experimental setup is shown in Fig 7a.

The comparison between the measured results and the theoretical model is presented in Fig 7b. The root mean squared errors (RMSE) of the estimated rotation angles for the three measurements were 0.025 rad, 0.038 rad, and 0.026 rad, respectively. In obtaining the model values, the corresponding Θ was selected using the aforementioned sampling method for the measured *Z*. The small RMSE values confirmed the validity of the model.

### Robot motions

With the implementation of the design and control strategy explained in the preceding sections, MOFU was able to independently expand and contract and perform rotational motions using the differential two-wheel drive. During an expansion–contraction motion with a vertical displacement of 65 mm, the robot base rotated by up to $28.6^{\circ }$, enabling evaluation of the two types of motion inherent to the Jitterbug structure—expansion–contraction and base rotation. The control parameters used for the expansion–contraction motion are summarized in Table 3.

**Table 3:** Control parameters for the expansion–contraction motion.

Parameter	Value
Period	6 s
Motor rotation amplitude	7π rad (3.5 revolutions)
Control frequency	10 Hz

The inverse function of the model is complex; hence, as mentioned earlier, the applicable range of the theoretical model (0–1.0 rad) was sampled at 45 points, and a correspondence table was created for motor control. This method enabled compensation for rotational motion induced by expansion–contraction, facilitating four motion conditions by combining the presence or absence of expansion–contraction and rotational motion. These conditions were used in the impression evaluation experiments described in the next section.

### Methods

In this study, we conducted three experiments involving different participants to investigate the effect of whole-body expansion–contraction movements, which are volumetric motions, on the perception of animacy in robots.

- **Experiment 1 (Single-robot condition):** Participants evaluated a single robot in a stationary state, under conditions with and without expansion–contraction and rotational motions.
- **Experiment 2 (Dual-robot condition):** Participants assessed their impressions when two robots were simultaneously displayed, both with and without expansion–contraction movements.
- **Experiment 3 (Locomotion condition):** Participants assessed the animacy of the robot when presented with locomotion alone or locomotion combined with rotational and expansion–contraction motions.

These experiments were designed to test the following hypotheses:

1. Expansion–contraction increases perceived animacy compared with stationary or rotational motions alone.
2. Presenting two robots simultaneously increases perceived animacy compared with presenting a single robot.
3. Combining rotational and expansion–contraction motions with locomotion enhances animacy perception compared with locomotion alone.

In addition to the a priori hypotheses, Experiment 2 included the same four motion conditions as Experiment 1 but with two robots, to examine generalization. Comparisons among these four dual-robot conditions were treated as exploratory analyses and are reported separately from the a priori predictions. The confirmatory test for the second prediction compares EC with one robot versus EC with two robots.

### Participants

We recruited participants online through a crowdsourcing platform (Yahoo! Crowdsourcing). Recruitment was conducted in a single round for all three experiments (Experiment 1, Experiment 2, and Experiment 3), with participants randomly assigned to each experimental condition.

Multiple participation across experiments was prohibited by the platform. In total, 552 participants took part, of whom 54 were excluded due to incomplete or invalid responses, leaving 498 valid participants (353 male, 140 female, 5 preferred not to answer; *M* = 51.69, *SD* = 11.67 years). We provide information about the number of participants allocated to each experiment and the sample sizes that were calculated in advance in the following subsections. For all statistical analyses conducted in this study, the significance level was set at α=0.050.

**Ethics statement.** The study protocol was approved by the Ethics Committee of the University of Electro-Communications (No. H23064(4)) and by the Sony Bioethics Committee (No. 23-R-0040). All participants provided written informed consent before participating in the experiment. The online questionnaire was released on December 3, 2024, and responses were collected and finalized on the same day.

#### Experiment 1 (Single-robot condition).

The required sample size for this experiment was calculated using G*Power 3.1 software [25]. An a priori power analysis was conducted for the planned 2×2 between-subjects ANOVA. Because the primary confirmatory question concerned whether expansion–contraction affected animacy ratings, the calculation targeted a planned single-degree-of-freedom ANOVA effect. A medium effect size (*f* = 0.25), significance level α=0.050, and statistical power 1−β=0.80 were assumed. The following parameters were specified: numerator *df* = 1, denominator df=N−4 (accounting for four groups), and number of groups =2×2=4. This calculation should be interpreted as providing power for the specified one-degree-of-freedom effect, not as a guarantee of sufficient power for all main effects and the interaction simultaneously. The analysis indicated that a minimum of *N* = 128 participants was required. However, a larger number of participants was recruited in anticipation of invalid responses, resulting in a final valid sample of *N* = 210 participants. Of these, *n* = 155 were male, *n* = 54 were female and *n* = 1 was a participant who chose not to report their gender, with a mean age of *M* = 51.26, *SD* = 12.04 years. These data were used for analysis.

#### Experiment 2 (Dual-robot condition).

The required sample size for this experiment was calculated using G*Power 3.1 software [25]. For consistency with Experiment 1, the target sample size for the four dual-robot motion conditions was set using the same planned single-degree-of-freedom ANOVA effect calculation in a 2×2 between-subjects design. This analysis targeted one specified effect and was not intended to provide simultaneous power for all main effects and the interaction. The analysis indicated that a minimum of *N* = 128 participants was required. However, a larger sample was recruited to anticipate invalid responses, resulting in a final valid sample of *N* = 207 participants. The comparison between the one-robot and two-robot EC conditions was treated as the pre-planned confirmatory comparison and analyzed separately using an independent-samples *t*-test; comparisons among the four dual-robo*t* motion conditions were exploratory. Of these, *n* = 147 were male and *n* = 58 were female and *n* = 2 were participants who chose not to report their gender, with a mean age of *M* = 52.19, *SD* = 12.16 years. These data were used for analysis.

#### Experiment 3 (Locomotion condition).

The sample size for this experiment was determined using G*Power 3.1 software [25]. A priori power analysis was conducted assuming an independent-samples *t* test (two-tailed), with a medium effect size (Cohen’s *d* = 0.50), significance level α=0.050, and statistical power 1−β=0.80. The group size allocation ratio was set to 1 (equal sample sizes). Based on these parameters, the required sample size was calculated to be *N* = 128 (64 per group). The analysis yielded *df* = 126, a critical *t* value of 1.98, a noncentrality parameter δ=2.83, and an actual power of 0.801, indicating that a*t* least 128 participants were necessary to ensure sufficient statistical power. However, as platform constraints necessitated an equal distribution of participants across all experimental conditions, the final valid sample size fell short of the target. After excluding invalid responses, the dataset comprised *N* = 81 participants (male *n* = 51, female *n* = 28, undisclosed *n* = 2), with a mean age of *M* = 51.57 years and a standard deviation of *SD* = 9.26 years. These data were used for analysis. The implications of the reduced sample size are addressed in the Limitations section.

### Materials

The experiments employed videos of MOFU’s movements, introduced in the preceding section, as stimuli. The experimental conditions were meticulously crafted by seamlessly integrating the motions generated by the Jitterbug structure with the differential two-wheel drive.

Each video, lasting 20 s, commenced at 0 s with the robot in a contracted state. At 15 s, a keyword appeared in the lower-right corner of the screen to confirm participant engagement. The video concluded at 20 s. Below are the details of each motion. Corresponding videos of each movement are publicly available in Figshare at https://doi.org/10.6084/m9.figshare.30437177. Each video file is named according to the experimental condition it represents. Conditions labeled “(2 robots)” in the manuscript are indicated as “×2” in the video filenames.

The robot motion conditions used in the three experiments are summarized in Table 4. In Experiment 1, a single robot was presented, and four conditions were compared by combining the presence or absence of rotation and expansion–contraction. The conditions and their abbreviations are as follows:

- **NBM (No Body Motion)**: Neither rotation nor expansion–contraction.
- **RM (Rotational Motion)**: Rotation only (implemented by the differential two-wheel drive).
- **EC (Expansion–Contraction)**: Expansion–contraction only (achieved by canceling the rotational component generated by the Jitterbug structure using the differential two-wheel drive).
- **RM + EC (Rotational Motion + Expansion–Contraction)**: Rotation combined with expansion–contraction, realized solely by the Jitterbug structure.

**Table 4:** List of MOFU motion conditions. Abbreviations: NBM = No Body Motion, RM = Rotational Motion, EC = Expansion–Contraction (Exp.–Cont.), LOC = Locomotion. Note that comparisons among the four dual-robot motion conditions were exploratory and are reported separately from the confirmatory test of the two- versus one-robot comparison under EC.

Conditions	Robots	Rotation	Exp.–Cont.	Locomotion
NBM	1	–	–	–
RM	1	✓	–	–
EC	1	–	✓	–
RM + EC	1	✓	✓	–
NBM (2 robots)	2	–	–	–
RM (2 robots)	2	✓	–	–
EC (2 robots)	2	–	✓	–
RM + EC (2 robots)	2	✓	✓	–
LOC	1	–	–	✓
LOC + RM + EC	1	✓	✓	✓

In Experiment 2, the conditions were identical to those in Experiment 1, except that two robots were presented instead of one (denoted as “(2 robots)” after each condition name).

In Experiment 3, locomotion was introduced, and two conditions were compared: LOC (locomotion alone) and LOC + RM + EC (locomotion combined with rotational and expansion–contraction motions).

Animacy was assessed using the Animacy subscale (Series II) of the Godspeed Questionnaire Series [26]. This scale is available in multiple languages [27]. However, as all participants in this study were Japanese speakers, they responded to the Japanese version of the questionnaire. Responses were collected on a 5-point Likert-type scale. It should be noted that the Godspeed Questionnaire Series, including its Animacy subscale, has been criticized for psychometric limitations, including concerns about construct validity and reliability across contexts; for a detailed overview, see Saad et al. [28]. Ho and MacDorman [29] reported overlap between the Godspeed Animacy subscale and other indices, indicating that Godspeed-based measures may not sufficiently separate theoretically distinct perceptual dimensions. Carpinella et al. [30] developed the Robotic Social Attributes Scale (RoSAS) as a validated measure of robot social attributes. Nevertheless, the Godspeed Questionnaire Series remains one of the most widely used instruments for evaluating human perceptions of robots in human–robot interaction research. Therefore, the Godspeed Animacy subscale should be interpreted in this study as one operationalized measure of perceived animacy, rather than as a comprehensive psychometric account of robot social perception. The scale captures differences in perceived animacy along a continuum of degree, rather than providing a binary measure of the presence or absence of animacy.

Participants were also required to answer a multiple-choice question (with 10 options) about a keyword presented in the latter part of the video to prevent satisficing in the online survey. Additionally, an instruction check item was appended to the end of the Godspeed Questionnaire Series as an attention check to detect satisficing responses. In this item, participants were explicitly instructed to select the middle option of the scale (e.g., “For this question, please choose the middle option”), allowing inattentive responses to be identified. Furthermore, participants had the option to provide free-text comments about the robot’s movements. These comments were collected only as optional open feedback and were not included in the analyses reported in this study.

#### Experiment 1 (Single-robot condition).

In Experiment 1, participants were presented with four conditions: NBM, RM, EC, and RM + EC. All robot movements followed a triangular waveform with a 6 s period. NBM represented the stationary state. RM reproduced only the rotational component generated by the Jitterbug structure using the differential two-wheel drive. EC presented expansion–contraction alone, with the rotational component inherent in the Jitterbug structure canceled out by the differential drive. RM + EC represented the condition in which both expansion–contraction and rotation occurred simultaneously through the Jitterbug structure.

#### Experiment 2 (Dual-robot condition).

In Experiment 2, two robots were presented under the same four conditions as in Experiment 1: NBM (2 robots), RM (2 robots), EC (2 robots), and RM + EC (2 robots). The expansion–contraction period for each robot was randomly varied between 5 and 7 s. Reproducibility across videos was ensured by fixing the random seed so that the timing of expansion–contraction differed between the two robots in a reproducible manner. This manipulation prevented full synchronization of their movements. Comparisons among the four dual-robot conditions were pre-specified for descriptive characterization and treated as exploratory analyses. Additionally, Experiment 2 included a confirmatory test of the prediction that presenting two robots would increase perceived animacy compared with a single robot under the EC condition.

#### Experiment 3 (Locomotion condition).

In Experiment 3, participants were presented with two conditions: LOC and LOC + RM + EC. In both conditions, MOFU alternated between locomotion and stopping with a 2 s cycle, comprising 1.5 s of locomotion followed by 0.5 s of stopping. In the LOC + RM + EC condition, expansion–contraction occurred only during locomotion phases and was withheld during stops. The expansion and contraction phases alternated across locomotion cycles, beginning with expansion during the first cycle.

### Procedure

Participants provided informed consent via an online form and then watched a single 20-s video. After viewing the video, they answered a multiple-choice question (10 options) about a keyword appearing in the latter part of the video. Next, they completed the six items of the Animacy subscale of the Godspeed Questionnaire Series. An instruction check item was appended to the end of the questionnaire to assess attention (e.g., “For this question, please choose the middle option”). Participants were then given the option to provide free-text comments about the robot’s movements. Those who correctly answered the keyword question received a monetary reward of 20 yen. Each participant viewed only one video condition and could not participate in multiple conditions.

## Results

In this study, we investigated participants’ perceptions of animacy in response to the movements of MOFU, a robot capable of whole-body expansion and contraction. The findings from three experiments are presented below.

### Experiment 1 (Single-robot condition)

**Fig 8:**
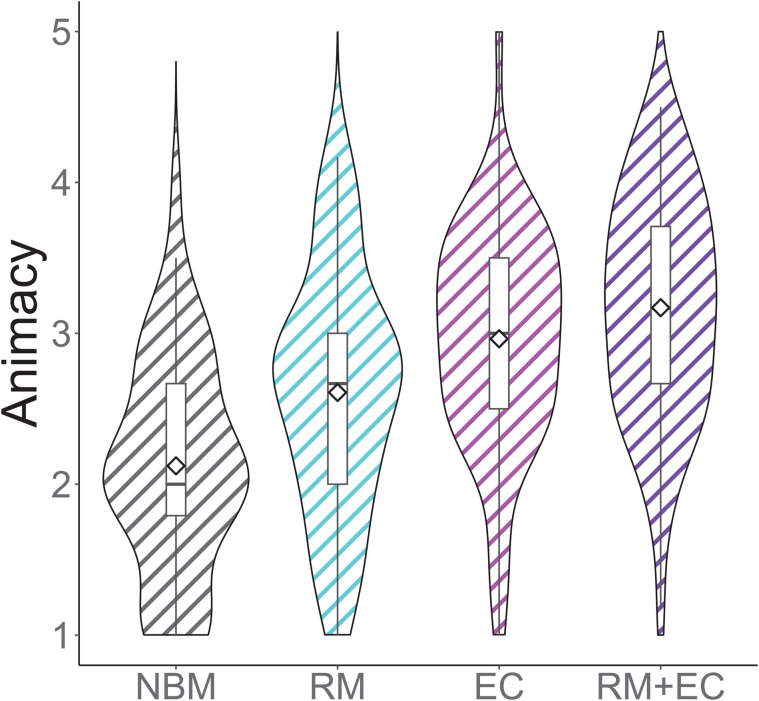
Comparison of animacy across the single-robot conditions. The y-axis shows the animacy score, computed for each participant as the mean of the six items of the Godspeed Animacy subscale (Series II), rated on a 1–5 Likert-type scale (1 = low, 5 = high). The boxes represent the distribution across participants (median, quartiles, and range). The mean value is indicated by a diamond marker.

Descriptive statistics showed that animacy scores were numerically higher in conditions with EC present (EC: *M* = 2.96, RM + EC: *M* = 3.17) than with EC absent (NBM: *M* = 2.12, RM: *M* = 2.61), and numerically higher with RM present (RM: *M* = 2.61, RM + EC: *M* = 3.17) than with RM absent (NBM: *M* = 2.12, EC: *M* = 2.96), consistent with a pattern of independent additive effects of both factors (Fig 8). The descriptive statistics for each condition are presented in Table 5.

**Table 5:** Descriptive statistics of animacy scores by condition in Experiment 1.

Condition	Participants	Mean	SD
NBM	52	2.12	0.72
RM	51	2.61	0.79
EC	55	2.96	0.74
RM + EC	52	3.17	0.75

Prior to statistical testing, assumptions were checked. Shapiro–Wilk tests indicated normality in all conditions (NBM: *W*(52) = 0.966, *p* = 0.137; RM: *W*(51) = 0.976, *p* = 0.375; EC: *W*(55) = 0.974, *p* = 0.289; RM + EC: *W*(52) = 0.979, *p* = 0.496). Levene’s test confirmed homogeneity of variance across conditions (*F*(3, 206) = 0.218, *p* = 0.884). Thus, the assumptions for ANOVA were met.

As a pre-planned confirmatory analysis, a two-way ANOVA was conducted to investigate the effects of rotational motion, expansion–contraction, and their interaction. Details of the ANOVA are shown in Table 6. The main effect of the rotational motion was significant, *F*(1, 206) = 11.236, *p* < 0.001. The main effect of expansion–contraction was also significant, *F*(1, 206) = 46.208, *p* < 0.001. In contrast, the interaction between the rotational motion and expansion–contraction was not significant, *F*(1, 206) = 1.836, *p* = 0.177.

**Table 6:** Results of the two-way ANOVA in Experiment 1.

Source	Sum Sq.	df	Mean Square	F	Sig.	$\eta_{𝐩}^{2}$
Corrected Model	33.17	3	11.06	19.766	<0.001	0.22
Intercept	1547.62	1	1547.62	2766.753	<0.001	–
RM	6.29	1	6.29	11.236	0.001	0.05
EC	25.85	1	25.85	46.208	<0.001	0.18
RM×EC	1.03	1	1.03	1.836	0.177	0.01
Error	115.23	206	0.559			
Total	1696.02					
Corrected Total	148.40	209				

The significant main effect of RM indicates that, averaged across EC conditions, rotational motion led to higher animacy ratings than no rotation. The significant main effect of EC indicates that, averaged across RM conditions, expansion–contraction led to higher animacy ratings than no expansion–contraction. The non-significant interaction is consistent with these two effects being additive: EC promoted animacy regardless of whether RM was present or not. These results provide direct confirmatory evidence that both RM and EC independently boosted perceived animacy ratings.

As an additional comparison between the single-motion conditions, Tukey’s HSD test revealed no significant difference between the RM-only and EC-only conditions ($p_{adj}=0.072$), suggesting that both motion types produced comparable increases in animacy ratings in this paradigm.

### Experiment 2 (Dual-robot condition)

As noted in the Methods section, the comparison between the one-robot and two-robot EC conditions was the pre-planned confirmatory comparison. The subsequent comparison among the four dual-robot motion conditions was exploratory and is reported to describe the pattern of responses across motion types.

**Fig 9:**
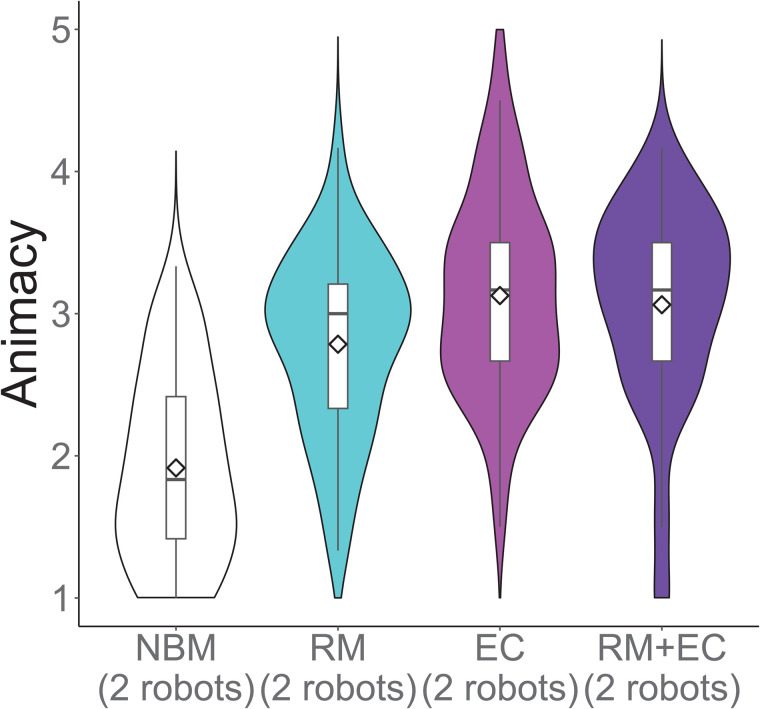
(Exploratory) Comparison of animacy across the dual-robot conditions. The y-axis shows the animacy score, computed for each participant as the mean of the six items of the Godspeed Animacy subscale (Series II), rated on a 1–5 Likert-type scale (1 = low, 5 = high). The boxes represent the distribution across participants (median, quartiles, and range). The mean value is indicated by a diamond marker.

**Exploratory analyses.** The following analyses compared animacy ratings among the four dual-robot conditions. The median animacy score was lowest in the NBM (2 robots) condition, followed by the RM (2 robots) condition, while the RM + EC (2 robots) and EC (2 robots) conditions showed identical median values (Fig 9). The descriptive statistics for each condition are listed in Table 7.

**Table 7:** Descriptive statistics of animacy scores by condition in Experiment 2.

Condition	Participants	Median	IQR
NBM (2 robots)	51	1.83	1.00
RM (2 robots)	52	3.00	0.88
EC (2 robots)	51	3.17	0.83
RM + EC (2 robots)	53	3.17	0.83

Shapiro–Wilk tests were performed to ensure the validity of the statistical test. The assumption of normality was rejected for the NBM (2 robots) condition (*W*(51) = 0.942, *p* = 0.015) and the RM + EC (2 robots) condition (*W*(53) = 0.918, *p* = 0.001). In contrast, normality was confirmed for the RM (2 robots) condition (*W*(52) = 0.968, *p* = 0.176) and the EC (2 robots) condition (*W*(51) = 0.986, *p* = 0.789). After applying Holm’s correction, the violation of normality remained for NBM (2 robots) ($p_{adj}=0.045$) and RM + EC (2 robots) ($p_{adj}=0.006$), whereas normality was retained for RM (2 robots) ($p_{adj}=0.353$) and EC (2 robots) ($p_{adj}=0.789$).

As the assumption of normality was not met in certain conditions, a non-parametric Kruskal–Wallis test was conducted. The results revealed a significant difference among conditions, $\chi^{2}(3)=66.844,p<0.001$. Post hoc pairwise comparisons using Dunn’s test with Bonferroni correction revealed that the key distinction was between the no-motion baseline and all motion conditions: NBM (2 robots) was rated significantly lower than RM (2 robots) ($Z=-5.047,p_{adj}<0.001$), EC (2 robots) ($Z=-7.030,p_{adj}<0.001$), and RM + EC (2 robots) ($Z=-7.139,p_{adj}<0.001$). In contrast, no significant differences were found among the three motion conditions (all $p_{adj}>0.10$), indicating that the type of motion (RM, EC, or their combination) did not differentially affect animacy ratings when two robots were presented.

**Fig 10:**
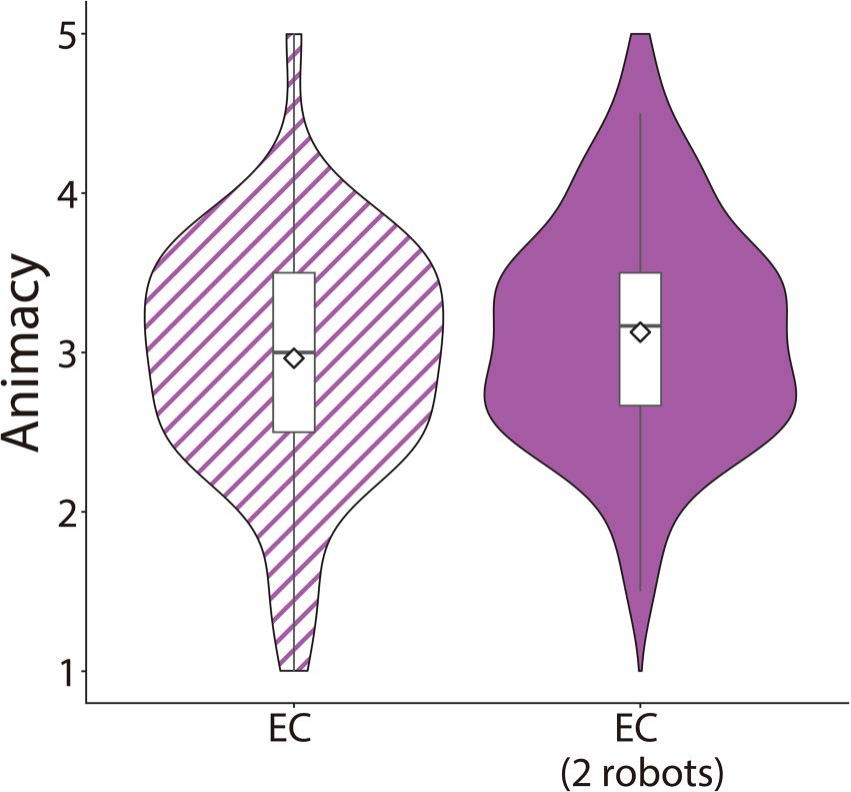
Comparison of animacy between single-robot and dual-robot EC conditions. The y-axis shows the animacy score, computed for each participant as the mean of the six items of the Godspeed Animacy subscale (Series II), rated on a 1–5 Likert-type scale (1 = low, 5 = high). The boxes represent the distribution across participants (median, quartiles, and range). The mean value is indicated by a diamond marker.

**Confirmatory analysis.** As a pre-planned confirmatory test, we compared the EC condition with EC (2 robots) (Fig 10). Levene’s test indicated no significant difference in variances (*F*(1, 104) = 0.008, *p* = 0.929). An independent-samples *t*-test assuming equal variances revealed no significant difference between the two conditions, t(104)=−1.173,p=0.244. The 95% confidence interval was [−0.441, 0.113], indicating that the mean difference between the two conditions was not statistically significant. These results do not support the hypothesis that presenting two robots simultaneously increases perceived animacy compared with a single robot.

### Experiment 3 (Locomotion condition)

**Fig 11:**
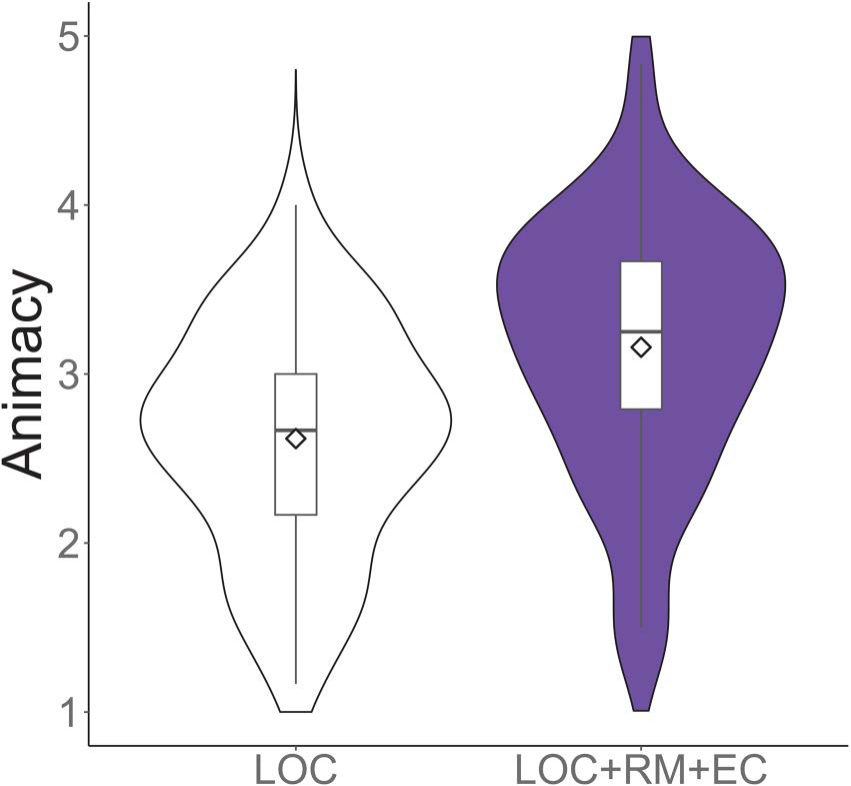
Comparison of animacy across the locomotion conditions. The y-axis shows the animacy score, computed for each participant as the mean of the six items of the Godspeed Animacy subscale (Series II), rated on a 1–5 Likert-type scale (1 = low, 5 = high). The boxes represent the distribution across participants (median, quartiles, and range). The mean value is indicated by a diamond marker.

The mean animacy score was higher in the LOC + RM + EC condition than in the LOC condition (Fig 11). The descriptive statistics for each condition are presented in Table 8.

**Table 8:** Descriptive statistics of animacy scores by condition in Experiment 3.

Condition	Participants	Mean	SD
LOC + RM + EC	40	3.16	0.77
LOC	41	2.62	0.68

Shapiro–Wilk tests were conducted to check the assumptions for statistical testing. Normality was confirmed for both conditions (LOC + RM + EC: *W*(40) = 0.971, *p* = 0.389; LOC: *W*(41) = 0.977, *p* = 0.581). Levene’s test also confirmed homogeneity of variance between the two conditions (*F*(1, 79) = 0.412, *p* = 0.523).

As a pre-planned confirmatory analysis, an independent-samples *t*-test was performed. The results showed that the mean animacy score in the LOC + RM + EC condition (*M* = 3.158) was significantly higher than that in the LOC condition (*M* = 2.618), *t*(79) = 3.359, *p* = 0.001. The 95% confidence interval for the difference between the two means was [0.220, 0.861], indicating a statistically significant difference. This suggests that combining rotational and expansion–contraction motions with locomotion significantly enhances the perceived animacy of MOFU.

## Discussion

In this study, we examined the influence of volumetric motion, specifically expansion–contraction, on the perception of animacy in robots, using the physical robot MOFU. MOFU employs a Jitterbug mechanism, enabling whole-body expansion and contraction with a simple configuration, and its movements were used to evaluate animacy impressions. Here, we discuss the interpretation of the results, limitations of the study design, and directions for future research.

The results of this study indicate that both expansion–contraction (EC) and rotational motion (RM) in the Jitterbug structure significantly influenced animacy perception, while their interaction was not significant. Post hoc comparisons showed no significant difference between the EC and RM + EC conditions, suggesting that adding RM does not provide additional benefit when EC is present. Overall, both movements enhanced animacy perception, and EC and RM elicited comparable levels of animacy in this paradigm (no significant difference between them, $p_{adj}=0.072$).

In the multi-robot conditions, NBM (2 robots) consistently received lower animacy ratings than the other conditions (RM, EC, RM + EC). This suggests that the “type of movement,” such as rotation or expansion–contraction, may have a stronger influence on perceived animacy than the number of robots. Although we hypothesized that presenting two robots simultaneously would increase animacy ratings compared with a single robot, this prediction was not supported, as no significant difference was observed between single- and dual-robot presentations. Single-robot presentation may focus attention on “individual movement,” whereas multi-robot presentation could raise expectations for “inter-robot relationships” or “interactions.” However, in this study, no elements suggesting coordination or competition between robots were designed, and the movement sequences were set independently.

In addition, animacy ratings were higher when rotational and expansion–contraction motions were combined with locomotion than when locomotion occurred alone. Parovel et al. [16] reported that moving squares with area changes, such as caterpillar-like deformation, were perceived as more animate than squares undergoing simple linear motion. As MOFU also generates volumetric changes through expansion and contraction, these findings suggest that such movements may contribute to improving animacy ratings during robot locomotion; however, Experiment 3 did not isolate their contribution from that of rotational motion.

### Limitations

This study has several limitations. First, it focused on the presence or absence of expansion–contraction but did not directly compare it with other movement types, such as articulated or axial motion. Therefore, the specific contribution of expansion–contraction to enhancing animacy relative to other types of movement remains unclear. Additionally, parameters such as the cycle and amplitude of expansion–contraction, as well as MOFU’s external appearance, were not systematically examined.

Second, the multi-robot conditions only increased the number of robots presented, without introducing interactions between them. Future research should explore coordinated or competitive behaviors, which may elicit higher levels of perceived animacy.

Third, while the combination of rotational and expansion–contraction motions with locomotion was shown to enhance animacy, the effects of synchronization and motion parameters, such as speed, timing, and smoothness, were not analyzed. Precise control of these parameters could help identify the underlying perceptual mechanisms.

Fourth, this study employed an online video-based paradigm, which necessarily excluded tactile and auditory sensory channels. MOFU’s fur-like exterior and quiet operation are design features intended for direct physical interaction, and their contribution to animacy perception can only be assessed through in-person experiments. Furthermore, the specific impact of MOFU’s appearance on animacy perception was not examined in the present study. This includes possible effects of color: although blue and pink were used to distinguish the robots and to avoid associations with familiar animal species, these colors may also evoke gender-related social categorization.

Fifth, this study relied exclusively on the Godspeed Animacy subscale to operationalize animacy perception. Because animacy was the primary dependent variable, this measurement choice warrants caution regarding the broader generalizability of the findings. Prior work has noted overlap between the Godspeed Animacy subscale and other indices, and alternative measures of robot social attributes, such as RoSAS, have been developed. Thus, the present findings should be interpreted as differences in one operationalized measure of perceived animacy, rather than as a comprehensive psychometric account of robot social perception. Given that the paper’s main focus is social and therapeutic applications, additional constructs such as perceived comfort, warmth, eeriness, and approach intention were not assessed. High animacy scores do not necessarily translate to positive social presence or therapeutic value, and future studies should include a broader range of perceptual and affective measures.

Sixth, although both the RM and RM + EC conditions involve rotational motion, their mechanical implementations differ: the RM condition achieves rotation via wheel drive, whereas the RM + EC condition relies on rotation induced by the Jitterbug structure. Furthermore, the EC condition employs wheel drive to actively cancel the rotation induced by the Jitterbug mechanism. These differences in kinematic implementation—including potential differences in smoothness, oscillation, and stabilization artifacts—may have introduced confounds, limiting direct comparisons between conditions involving rotation. To examine potential differences in motion characteristics, we visualized the motor kinematics, including angular position, angular velocity, and angular acceleration, separately for the expansion–contraction motor and the rotational motor across conditions (S2 Fig). Future studies should control for these mechanical differences through more carefully designed experimental conditions.

Seventh, all three experiments employed a between-subjects design in which each participant was exposed to only one condition. Although this approach avoids order and carryover effects, it makes the study susceptible to individual differences in response styles, particularly on Likert-type scales, which may obscure subtle differences between conditions (e.g., between EC and RM).

Eighth, the achieved sample size in Experiment 3 (*N* = 81) fell short of the planned target (*N* = 128) owing to crowdsourcing platform constraints. Underpowered studies are known to risk overestimating effect sizes; consequently, the robustness of the LOC vs. LOC+RM+EC finding is lower than originally intended and should be replicated with an adequately powered sample.

Ninth, the participant sample predominantly comprised older males (mean age approximately 51 years), reflecting the demographic profile of the crowdsourcing platform used for recruitment. This limits the generalizability of the findings to other target populations for social and therapeutic robots, such as children or clinical therapy groups, whose responses to animacy cues may differ substantially from those of the current sample.

### Future directions

The findings of this study suggest that fundamental volumetric motion, such as expansion–contraction, can independently increase perceived animacy ratings, even without locomotion. This indicates that expansion–contraction alone may serve as an effective design feature for robots in medical and welfare contexts, where space is limited and quiet operation is often essential.

Furthermore, investigating expansion–contraction as a fundamental form of three-dimensional motion could pave the way for incorporating diverse volumetric changes observed in human gestures and animal behaviors into robotic designs. This approach may ultimately provide guidelines for creating robots capable of more intuitive and expressive interactions.

Future research should also conduct in-person evaluations to capture the multisensory experience of interacting with MOFU, including its tactile and auditory properties. A more comprehensive assessment incorporating measures such as comfort, perceived warmth, eeriness, and approach intention would provide a broader understanding of MOFU’s suitability for social and therapeutic applications.

## Conclusion

In this study, we investigated the effect of expansion–contraction movements (volumetric motion) on the perception of animacy in robots using a video-based survey with the physical robot MOFU (MOrphing Fluffy Unit). MOFU features a Jitterbug structure that enables whole-body expansion and contraction driven by a single linear actuator and is also equipped with a differential two-wheel drive for locomotion.

The experimental results showed, first, that both expansion–contraction and rotational motion significantly increased animacy ratings compared with a baseline without body motion, with no significant difference between them in the direct comparison. Second, presenting two robots without inter-robot coordination or interaction did not significantly increase animacy relative to presenting a single robot under the expansion–contraction condition, suggesting that merely adding more robots without introducing coordinated behaviors does not enhance animacy. Conditions without movement, including those lacking rotational and volumetric motion, were consistently rated lower, highlighting the importance of the “type of movement” in animacy perception. Third, combining rotational and expansion–contraction motions with locomotion significantly increased animacy ratings compared with locomotion alone. All experiments employed between-participants comparisons, meaning the findings reflect consistent tendencies across independent participant groups rather than direct within-subject contrasts.

These findings indicate that relatively simple volumetric changes, such as expansion and contraction, can effectively enhance the perception of animacy without the need for complex appearances or mechanisms.

## Supporting information

S1 Fig Internal components of MOFU. Photographs of the internal components used in the robot system: (a) KM-1S-M4021 (Keigan Inc.); (b) SMARTCOBY Pro SLIM (CIO Co., Ltd.); (c) Raspberry Pi Zero 2 W (Raspberry Pi Ltd.); (d) MTSPW1220 (MISUMI Group Inc.); (e) TK-04 (Igus K.K.).
(TIF)

S2 Fig Motor kinematics of the expansion–contraction and rotational motors across experimental conditions. Angular position, angular velocity, and angular acceleration were plotted separately for the expansion–contraction motor and the rotational motor.
(TIF)
